# Tertiary lymphoid structures in the primary tumor site of patients with cancer-associated myositis: A case–control study

**DOI:** 10.3389/fmed.2022.1066858

**Published:** 2023-01-04

**Authors:** Hiroko Kadota, Takahisa Gono, Shinobu Kunugi, Yuko Ota, Mitsuhiro Takeno, Masahiro Seike, Akira Shimizu, Masataka Kuwana

**Affiliations:** ^1^Department of Allergy and Rheumatology, Graduate School of Medicine, Nippon Medical School, Tokyo, Japan; ^2^Scleroderma/Myositis Center of Excellence, Nippon Medical School Hospital, Tokyo, Japan; ^3^Department of Analytic Human Pathology, Graduate School of Medicine, Nippon Medical School, Tokyo, Japan; ^4^Department of Allergy and Rheumatology, Nippon Medical School Musashi Kosugi Hospital, Kawasaki, Japan; ^5^Department of Pulmonary Medicine and Oncology, Graduate School of Medicine, Nippon Medical School, Tokyo, Japan

**Keywords:** B cells, cancer, dermatomyositis, follicular dendritic cells, follicular helper T cell, myositis, tertiary lymphoid structure, tumor-infiltrating lymphocyte

## Abstract

**Objective:**

To investigate histologic features of immunological components in the primary tumor site of patients with cancer-associated myositis (CAM) by focusing on tumor-infiltrating lymphocytes (TILs) and tertiary lymphoid structures (TLSs), which play major roles in antitumor immunity.

**Methods:**

Cancer-associated myositis patients were selected from the single-center idiopathic inflammatory myopathy cohort based on the availability of primary tumor specimens obtained before the introduction of immunomodulatory agents. Control cancer subjects without CAM were selected from the cancer tissue repository at a ratio of 1:2 matched for demographics and cancer characteristics of CAM cases. A series of immunohistochemical analyses was conducted using sequential tumor sections. TLS was defined as an ectopic lymphoid-like structure composed of DC-LAMP^+^ mature dendritic cells, CD23^+^ follicular dendritic cells (FDCs) and PNAd^+^ high endothelial venules. TLS distribution was classified into the tumor center, invasive margin, and peritumoral area.

**Results:**

Six CAM patients and 12 matched non-CAM controls were eligible for the study. There was no apparent difference in the density or distribution of TILs between the groups. TLSs were found in 3 CAM patients (50%) and 4 non-CAM controls (33%). TLSs were exclusively located at the tumor center or invasive margin in CAM cases but were mainly found in the peritumoral area in non-CAM controls. FDCs and class-switched B cells colocalized with follicular helper T cells were abundantly found in the germinal center-like area of TLSs from CAM patients compared with those from non-CAM controls.

**Conclusion:**

The adaptive immune response within TLSs in the primary tumor site might contribute to the pathogenic process of CAM.

## Introduction

Idiopathic inflammatory myopathies (IIMs) are a group of conditions that affect skeletal muscles as well as extramuscular systems, such as skin, joints, heart, and lungs (1). The pathophysiology of IIMs remains uncertain, but it involves autoimmune responses characterized by production of a series of autoantibodies and activation of autoreactive T cells (2, 3). Adult patients with IIMs, including polymyositis (PM) and dermatomyositis (DM), have a higher risk for developing cancer than the general population (4, 5). Cancer-associated myositis (CAM) is defined as IIMs developing within a few years before or after the diagnosis of cancer (6, 7). CAM is considered a paraneoplastic syndrome because of the temporal relationship of the occurrence of these conditions. This is supported by the improvement of IIMs in several cases after resection of cancer without immunosuppressive therapy (8). It has been proposed that antitumor immune responses play a role in developing CAM, but few data are available on the adaptive immune response in the primary tumor site in CAM patients.

Accumulating lines of evidence have demonstrated the remarkable efficacy of immune checkpoint inhibitors on a variety of cancer types (9), indicating the indispensable role of adaptive immune responses against tumor cells in protection from cancer. Tumor-infiltrating lymphocytes (TILs) are a primary driver of adaptive immune responses against cancer, and the distribution, clustering, and interplay of their components are critical for eliciting effective antitumor immune response (10). TILs are located in the cancer stroma as aggregates or within tertiary lymphoid structures (TLSs), which are ectopic secondary lymphoid formations in the non-lymphoid tissue (11). TLSs are found not only in cancer lesions but also in chronically inflamed tissue in patients with various autoimmune diseases, allograft rejection, and infection, indicating a primary role in eliciting and maintaining acquired immune responses against tumor antigens, autoantigens, alloantigens, and foreign antigens (12). In fact, the appearance of TLSs in cancer lesions is associated with better overall survival in patients with various types of cancer (13, 14). In this case–control study, histologic features of the primary tumor site of CAM patients were compared with those of non-CAM cancer controls matched for demographic and cancer characteristics, focusing on TILs and TLSs.

## Materials and methods

### Subjects

In this case–control study, patients with CAM were selected from the IIM database of the Scleroderma/Myositis Center of Excellence at Nippon Medical School Hospital, established in July 2014, based on the following criteria: (i) fulfillment of the 2017 European League against Rheumatism (EULAR)/American College of Rheumatology (ACR) classification criteria for IIMs (15); (ii) age at diagnosis ≥ 18 years; (iii) development of IIMs within 3 years before or after the diagnosis of cancer (6); (iv) resection of the primary tumor before treatment with any immunomodulatory agents, including glucocorticoids, immunosuppressants, cytotoxic anticancer agents, molecular targeted agents, and immune checkpoint inhibitors; and v) availability of the resected primary tumor tissue for histologic evaluation. Subclassification of the IIM patients into DM, amyopathic DM (ADM), immune-mediated necrotizing myopathy (IMNM) or PM, and inclusion body myositis was made according to the EULAR/ACR classification criteria (15). Control non-CAM cancer subjects were selected from the cancer tissue repository of the Nippon Medical School Hospital (Clinical Rebiopsy Bank), which collects surgical specimens of primary tumor lesions from patients with lung, stomach, colon, breast, or ovarian cancer (16), and matched for age at cancer diagnosis (± 10 years), gender, tumor type, tumor nodule metastasis (TNM) classification of cancer, and tumor histology with CAM cases in a 1:2 ratio. All non-CAM controls had no history of any autoimmune diseases listed by the American Association of Autoimmune-Related Diseases^1^ and no record of preoperative adjuvant chemotherapy. This study was approved by the Institutional Review Board of Nippon Medical School Hospital (approval number 28-12-680), and written informed consent was obtained from all subjects.

### Clinical data

Standard demographic and clinical information of CAM patients was retrieved from the IIM database. Information regarding cancer was additionally obtained by a retrospective chart review and included age at diagnosis of CAM, age at surgery for cancer, sex, subclassification of IIMs, tumor type, TNM classification (17), tumor histology, treatment for IIMs and cancer, and outcomes of IIMs and cancer at the latest visit. Myositis-specific autoantibodies (MSAs) and myositis-associated autoantibodies (MAAs) were identified by immunoprecipitation combined with immunoblots (18), RNA immunoprecipitation assay (19), and a commercially available enzyme-linked immunosorbent assay kit (20).

### Tissue staining

Paraffin-embedded 4 μm-thick sections were prepared from tumor blocks containing the boundary between cancer and non-cancer areas confirmed by a skilled pathologist (SK). Then, the sections were deparaffinized in xylene, rehydrated with a graded series of ethanol solutions, and subjected to hematoxylin and eosin (HE) staining. For immunohistochemical staining, the sections were used for antigen retrieval, treated with absolute methanol containing 0.3% hydrogen peroxidase for 10 min to block endogenous peroxidase activity, and incubated for 1 h at room temperature or overnight at 4°C with one of the primary antibodies, including anti-CD3, anti-CD4, anti-CD8, anti-CD20, anti-dendritic cell lysosome-associated membrane glycoprotein (DC-LAMP), anti-CD23, anti-peripheral node addressin (PNAd), anti-CD138, anti-B-cell lymphoma 6 (BCL6), and anti-activation-induced cytidine deaminase (AID) antibodies (21). Supplementary Table 1 summarizes a list of the primary antibodies and antigen retrieval methods used for immunohistochemical analysis. The sections were subsequently incubated with a universal polymer containing peroxidase-conjugated goat anti-IgG Fab’ (Histofine^®^ Simple Stain PO(M) kit: Nichirei, Tokyo, Japan) (22), and the signals were visualized using 3,3’-diaminobenzidine (DAB). For the detection of PNAd, biotinylated anti-rat IgM antibodies (Abcam: Cambridge, UK) were employed, followed by incubation with peroxidase-labeled streptavidin. Mouse IgG1 or rabbit immunoglobulin (Dako Denmark A/S, Glostrup, Denmark) was used as a negative control. After counterstaining with hematoxylin, these slides were mounted and examined with an optical microscope (IX-81 inverted microscopes: Olympus, Tokyo, Japan), and images were recorded using the virtual slide system (SCN400F: Leica, Wetzlar, Germany).

Immunofluorescent double staining was performed according to published methods (23). Briefly, formalin-fixed and paraffin-embedded sections were deparaffinized, rehydrated, pretreated in Tris/ethylenediaminetetraacetic acid buffer (pH 9.0), and subjected to microwave treatment for 20 min. Non-specific binding sites were blocked with phosphate-buffered saline containing 5% bovine serum albumin for 60 min at room temperature, followed by incubation with different combinations of primary antibodies, including anti-CD4, anti-BCL6, and anti-AID antibodies, overnight at 4°C (Supplementary Table 1). The secondary antibodies used included goat anti-rabbit IgG-Alexa 488 and goat anti-mouse IgG-Alexa 568 (Abcam). Nuclei were counterstained with 4′,6-diamidine-2′-phenylindole dihydrochloride (DAPI; Thermo Fisher Scientific, Waltham, MA, USA). These slides were examined with a confocal laser fluorescence microscope (LSM 800 with Airyscan: ZEISS, Oberkochen, Germany), and images were recorded using a microscope digital camera (DP74; Olympus) with CellSens Dimension 2.3 software (Olympus).

### Histologic assessment

Slide images stored in the virtual slide system were used for the computerized quantitative assessment with the bioimage analysis software QuPath^2^ (24). After areas with crush, necrosis, hyalinization, non-specific staining, and artifacts generated during specimen preparation were excluded from the overall assessment (25), we defined the tissue region to be analyzed (simple tissue detection). The malignant tumor area was identified by a pathologist (SK) and was divided into three regions: invasive margin (IM), a 1 mm-wide area centered on the boundary between the cancer and non-cancer areas; central tumor (CT), the cancer area inside the IM; and peritumor (PT), the non-cancer area outside the IM (25). Next, all the cells in the area of interest were detected by using a built-in cell segmentation algorithm (cell detection), followed by classification of cell types and the presence or absence of staining (detection classifier). Finally, the number of stained immune cells (CD4/CD8/CD20) in the selected region was counted, and the density (cells/mm^2^) and the CD4/CD8 ratio were calculated. TILs were defined as lymphocytes infiltrated within the CT and IM areas (25).

TLSs were defined as an ectopic lymphoid-like structure detected in sequential sections that satisfied all of the following characteristics: (i) adjacent CD3^+^ T-cell and CD20^+^ B-cell areas; (ii) PNAd^+^ high endothelial venule (HEV) in and around the follicular structure; (iii) CD23^+^ follicular dendritic cells (FDCs) in the B-cell area; and (iv) DC-LAMP^+^ mature dendritic cells (DCs) in the T-cell area (26). TLSs with clear boundaries of the T-cell and B-cell areas were regarded as well-organized TLSs (27). TLS-like structures were defined as lymphocyte aggregation areas of ≥ 7,000 μm^2^ that did not meet the criteria for TLS (28). In addition, TLS-like structures were further classified into PNAd^+^, CD23^+^, and DC-LAMP^+^ TLS-like structures based on satisfaction of a criterion for PNAd^+^ HEV, CD23^+^ FDCs, or DC-LAMP^+^ mature DCs, respectively. The numbers of TLSs and TLS-like structures were counted in whole sections from individual subjects. We also assessed the density (cells/mm^2^), distribution (IM/CT/PT), maturity (well-organized/total TLS [%]), and mean diameter (mm^2^) of TLSs in individual subjects. The density of CD4^+^ T cells, CD8^+^ T cells, CD20^+^ B cells, AID^+^ cells, CD23^+^ FDCs, PNAd^+^ HEV, CD138^+^ plasma cells, and CD4^+^BCL6^+^ T follicular helper (Tfh) cells within the TLS was also evaluated and is shown in cells/mm^2^ except for CD23^+^ FDCs, which are shown as percentages.

### Statistical analysis

Continuous variables are shown as the mean ± standard deviation (SD) or median and range, as appropriate. A two-sided t test or Mann–Whitney U test was used to compare parametric and non-parametric variables, respectively. All statistical analyses were performed using the software program R (R version 4.1.3).

## Results

### Patient characteristics

Of 160 patients registered in the IIM database, 35 patients were classified as having CAM. The primary tumor specimen was available in 9 patients. After exclusion of 3 patients who received immunomodulatory agents before surgery, 6 patients with CAM were eligible for this study (Supplementary Figure 1). Twelve cancer patients without CAM who were matched for age at cancer diagnosis, sex, tumor type, TNM classification, and tumor histology were selected from the Clinical Rebiopsy Bank as non-CAM controls. The clinical characteristics of the 6 patients with CAM are shown in Table 1. All but one had classic DM or ADM, and the remaining patient was diagnosed with IMNM. The mean age at the time of diagnosis of IIM was 66.0 ± 8.7 years, and the median period from the diagnosis of IIM to cancer resection surgery was −3.5 months (range, −31 to 0). Two patients were positive for anti-TIF1-γ antibody, and anti-PL-12, anti-NXP2, and anti-SRP antibodies were positive in one patient each. The primary cancer sites included the stomach in 2 patients and the breast, lung, colorectum, and ovary in one patient each. The histological type was adenocarcinoma in all patients. Only one died of colorectal cancer 7 months after cancer diagnosis. The clinical characteristics of the 12 non-CAM controls are shown in Supplementary Table 2.

**TABLE 1:** Clinical characteristics of 6 patients with CAM.

Characteristics	CAM #1	CAM #2	CAM #3	CAM #4	CAM #5	CAM #6
Age at IIM diagnosis, years	69	65	62	77	73	50
Age at cancer resection surgery, years	69	64	62	74	73	49
Gender	Male	Female	Female	Male	Female	Female
IIM sub-classification	ADM	ADM	Classic DM	Classic DM	IMNM	Classic DM
MSAs/MAAs	Anti-PL-12	Negative	Anti-TIF1-γ	Anti-NXP2	Anti-SRP	Anti-TIF1-γ
Months from IIM diagnosis to surgery	0	−18	0	−31	−1	−6
Primary cancer site	Stomach	Stomach	Breast	Lung	Colorectum	Ovary
Cancer histological type	Adeno- carcinoma	Adeno- carcinoma	Adeno- carcinoma	Adeno- carcinoma	Adeno- carcinoma	Adeno- carcinoma
TNM classification	pT1b2 (SM2)	pT1N0M0	pT1N1aM0	pT2aN0M0	pT3aN0M0	pT3cNxM1b
Months from cancer diagnosis to last observation	186	76	157	49	7	23
Outcome at last observation	Alive	Alive	Alive	Alive	Dead	Alive

CAM, cancer-associated myositis; ADM, amyopathic dermatomyositis; DM, dermatomyositis; IMNM, immune-mediated necrotizing myopathy; MSA, myositis-specific antibody; MAA, myositis-associated antibody; TIF1-γ, transcriptional intermediary factor 1-γ; NXP2, nuclear matrix protein 2; SRP, anti-signal recognition particle; TNM: tumor-node-metastasis.

### TILs in CAM and non-CAM controls

**FIGURE 1:**
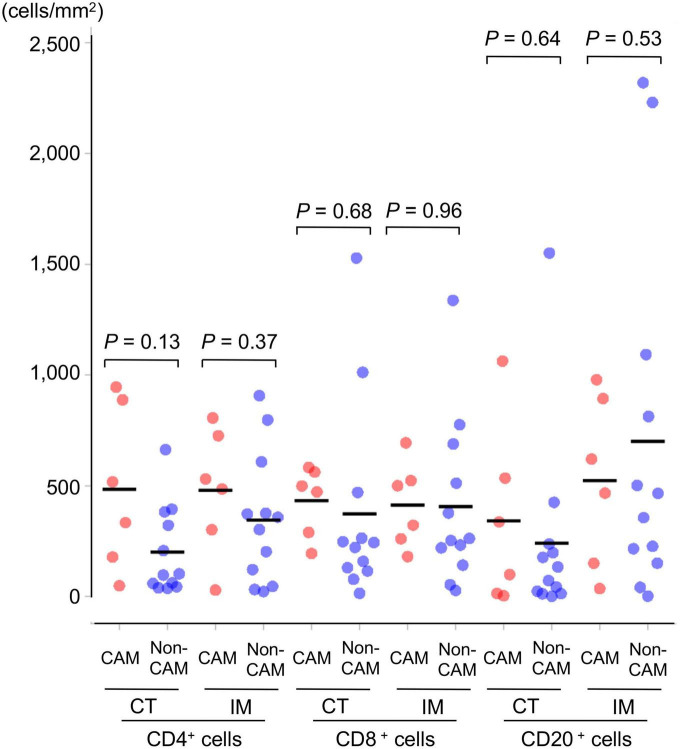
Density of TILs in 6 CAM patients and 12 non-CAM controls. The density of tumor-infiltrating CD4^+^ T cells, CD8^+^ T cells, and CD20^+^ B cells in the CT and IM areas of the cancer tissue was analyzed by computerized quantitative assessment using the bioimage analysis software QuPath. Horizontal lines denote the mean. CAM, cancer-associated myositis; TILs, tumor-infiltrating lymphocytes; CT, central tumor; IM, invasive margin.

The density of CD4^+^ T cells, CD8^+^ T cells, and CD20^+^ B cells in the CT and IM areas of the cancer tissue was similar between the 6 CAM patients and 12 non-CAM patients (Figure 1). CD4^+^ T cells in the CT and IM areas tended to be increased in CAM patients compared with non-CAM patients (*P* = 0.13 and 0.37, respectively), but the difference did not reach statistical significance, probably due to the small number of patients and a large variation among subjects.

### TLS in CAM and non-CAM controls

**FIGURE 2:**
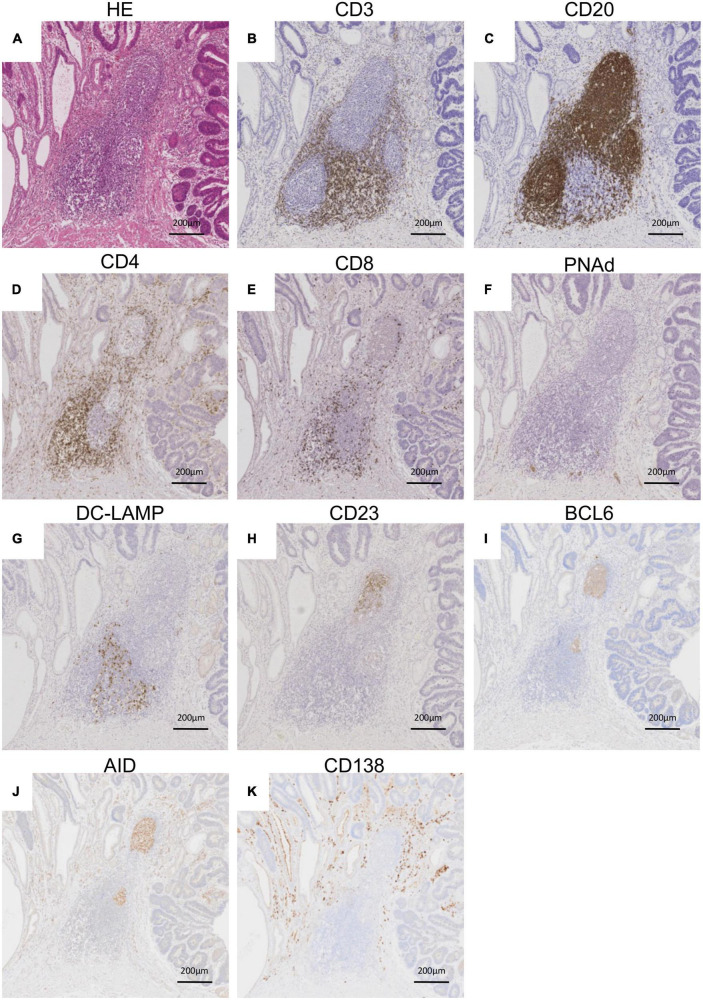
Representative histological features of TLS. Serial sections of the stomach cancer tissue from a representative CAM patient (CAM #2) were subjected to HE staining **(A)** and immunohistochemical staining for CD3 **(B)**, CD20 **(C)** CD4 **(D)**, CD8 **(E)**, PNAd **(F)**, DC-LAMP **(G)**, CD23 **(H)**, BCL6 **(I)**, AID **(J)**, and CD138 **(K)**. Scale bar = 200 μm. TLSs, tertiary lymphoid structures; HE, hematoxylin and eosin; PNAd, peripheral node addressin; DC-LAMP, dendritic cell lysosomal associated membrane glycoprotein; BCL6, B-cell lymphoma 6; AID, activation induced deaminase.

**FIGURE 3:**
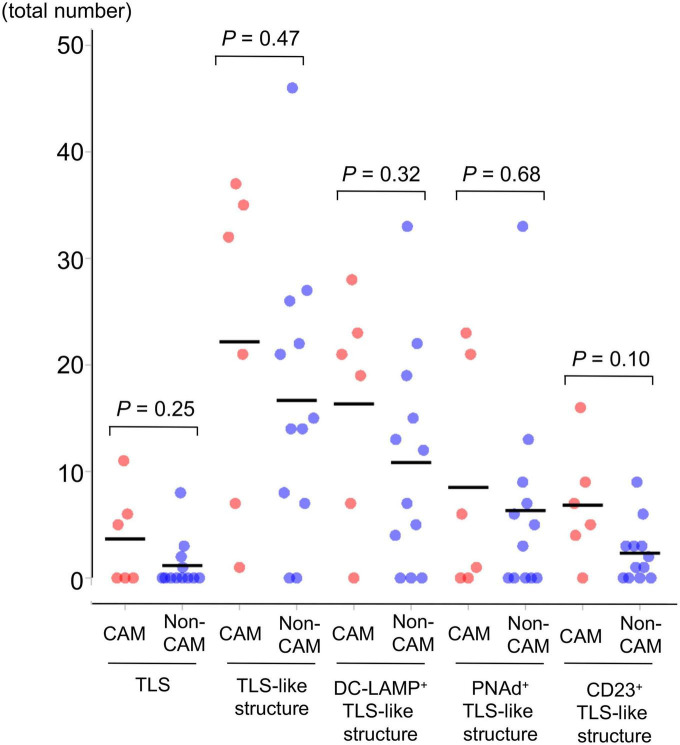
The number of TLSs and TLS-like structures in 6 CAM patients and 12 non-CAM controls. The number of TLSs, TLS-like structures, DC-LAMP^+^ TLS-like structures, PNAd^+^ TLS-like structures, and CD23^+^ TLS-like structures was analyzed by computerized quantitative assessment using the bioimage analysis software QuPath. The horizontal lines denote the mean. TLSs, tertiary lymphoid structures; TIL, tumor-infiltrating lymphocyte; CAM, cancer-associated myositis; DC-LAMP, dendritic cell lysosomal associated membrane glycoprotein; PNAd, peripheral node addressin.

Representative histological findings of TLSs found in CAM #2 are shown in Figure 2. A secondary lymphoid-like formation was easily identified by HE staining. A typical histologic characteristic of TLSs is a lymphoid follicle composed of an inner zone of CD20^+^ B cells (B-cell zone) and surrounding CD3^+^ T cells, mainly consisting of CD4^+^ T cells (T-cell zone). PNAd^+^ HEV and DC-LAMP^+^ mature DCs were predominantly localized in the T-cell zone. A germinal center (GC)-like structure, which was defined by the presence of CD23^+^ FDCs, formed a tight network in the B-cell zone within the follicle-like structure. Moreover, subgroups of lymphocytes in the GC-like structure expressed BCL6 or AID. CD138^+^ plasma cells were detected around TLSs in the stroma of the tumor tissue. Typical TLSs were detected in the tumor tissue of 3 (50%) of 6 CAM patients and 4 (33%) of 12 non-CAM controls. The number of TLSs and TLS-like structures in CAM patients and non-CAM controls is shown in Figure 3. There was a trend toward increased TLSs and TLS-like structures, especially CD23^+^ TLS-like structures, in CAM patients compared with non-CAM controls, but there was no statistically significant difference.

**FIGURE 4:**
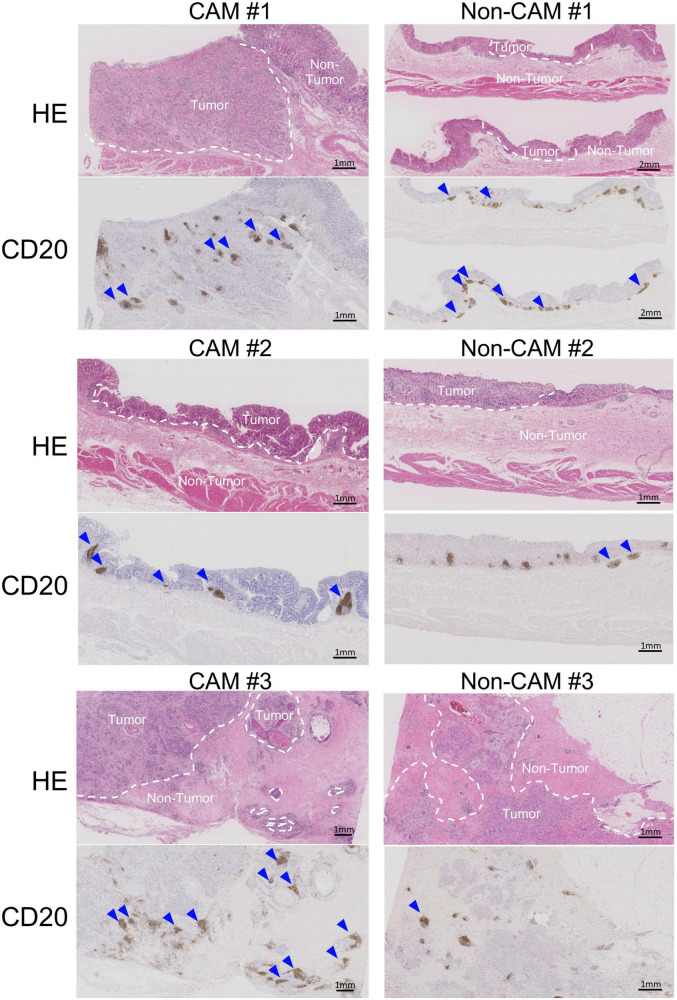
Low-magnification images of TLSs in 3 CAM patients and 3 non-CAM controls. HE staining and CD20 immunostaining were conducted in sequential tumor sections in 3 CAM patients (CAM #1-#3) and 3 non-CAM controls (non-CAM #1-#3). Broken lines in HE staining indicate the border between the tumor and non-tumor lesions, while blue arrows show TLSs with CD20^+^ B-cell aggregates. Scale bar = 1μm (2 μm in non-CAM #1) CAM, cancer-associated myositis; HE, hematoxylin and eosin.

To further investigate the detailed histological features of TLSs, a total of 20 TLSs detected in 3 CAM patients (two with gastric cancer and one with breast cancer) were compared with a total of 11 TLSs detected in 3 non-CAM controls matched for cancer type (Table 2). The total number and density of TLSs were numerically higher in CAM patients than in non-CAM controls. Low-magnification HE staining and CD20 staining of tumor sections in 3 CAM patients and 3 non-CAM controls revealed that TLSs were located exclusively in the CT or IM area in CAM patients, while TLSs were located mainly in the PT area in non-CAM controls (Figure 4). The maturity and size of TLSs were comparable between the groups.

**TABLE 2:** Histopathological features of TLSs in 3 CAM patients and 3 non-CAM controls.

Features	CAM			Non-CAM		
	#1	#2	#3	#1	#2	#3
Primary cancer site	Stomach	Stomach	Breast	Stomach	Stomach	Breast
Total number of TLSs	6	5	11	8	2	1
Density (/mm^2^)	0.049	0.047	0.025	0.029	0.014	0.0030
Distribution						
CT	3	0	0	0	0	0
IM	3	5	11	3	0	1
PT	0	0	0	5	2	0
Maturity (%)*	0	80	0	38	0	0
Size of TLSs (mm^2^)	0.10 ± 0.04	0.34 ± 0.13	0.34 ± 0.27	0.42 ± 0.34	0.10 ± 0.02	0.16

*Maturity is expressed as the proportion (%) of well-organized TLSs in total TLSs.

TLS, tertiary lymphoid structures; CAM, cancer-associated myositis; CT, central tumor; IM, invasive margin; PT, peritumor.

**FIGURE 5:**
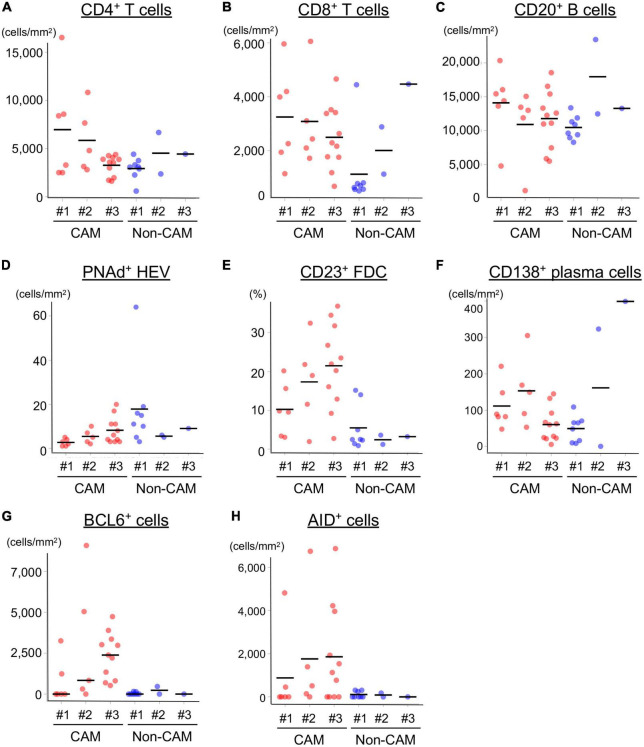
Density of immune cell components in TLSs from CAM patients and non-CAM controls. The density of CD4^+^ T cells **(A)**, CD8^+^ T cells **(B)**, CD20^+^ B cells **(C)**, PNAd^+^ HEV **(D)**, CD23^+^ FDCs **(E)**, CD138^+^ plasma cells **(F)**, BCL6^+^ cells **(G)**, and AID^+^ cells **(H)** in TLSs from 3 CAM patients and 3 non-CAM controls was analyzed by computerized quantitative assessment using the bioimage analysis software QuPath. The horizontal lines indicate the mean. TLSs, tertiary lymphoid structures; CAM, cancer-associated myositis; PNAd, peripheral node addressin; HEV, high endothelial venules; FDCs, follicular dendric cells; BCL6, B-cell lymphoma 6; AID, activation induced deaminase.

**FIGURE 6:**
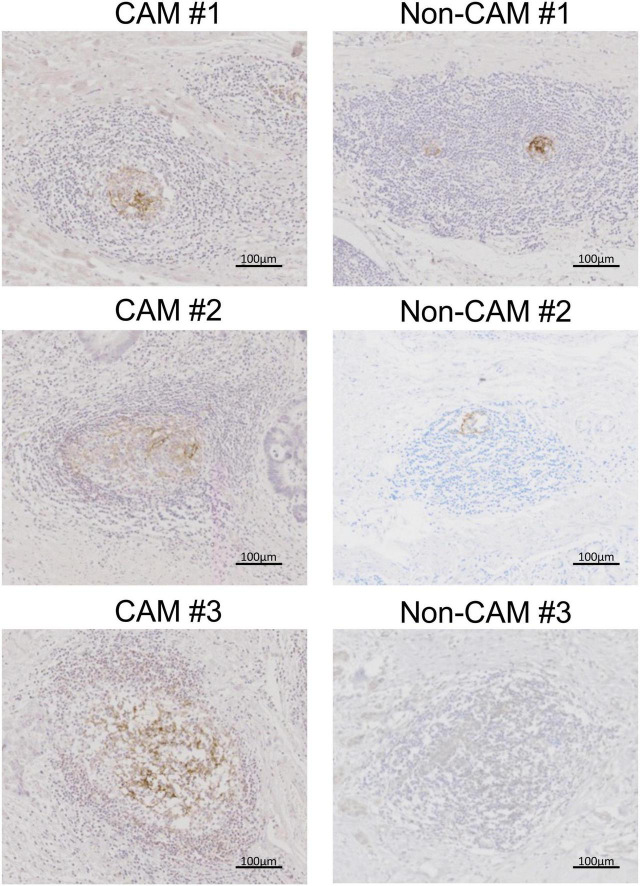
CD23^+^ FDCs in TLSs from CAM patients and non-CAM controls. CD23 immunostaining was conducted in representative TLSs from 3 CAM patients (CAM #1-#3) and 3 non-CAM controls (non-CAM #1-#3). Scale bar = 100 μm. FDCs, follicular dendric cells; TLSs, tertiary lymphoid structures; CAM, cancer-associated myositis.

**FIGURE 7:**
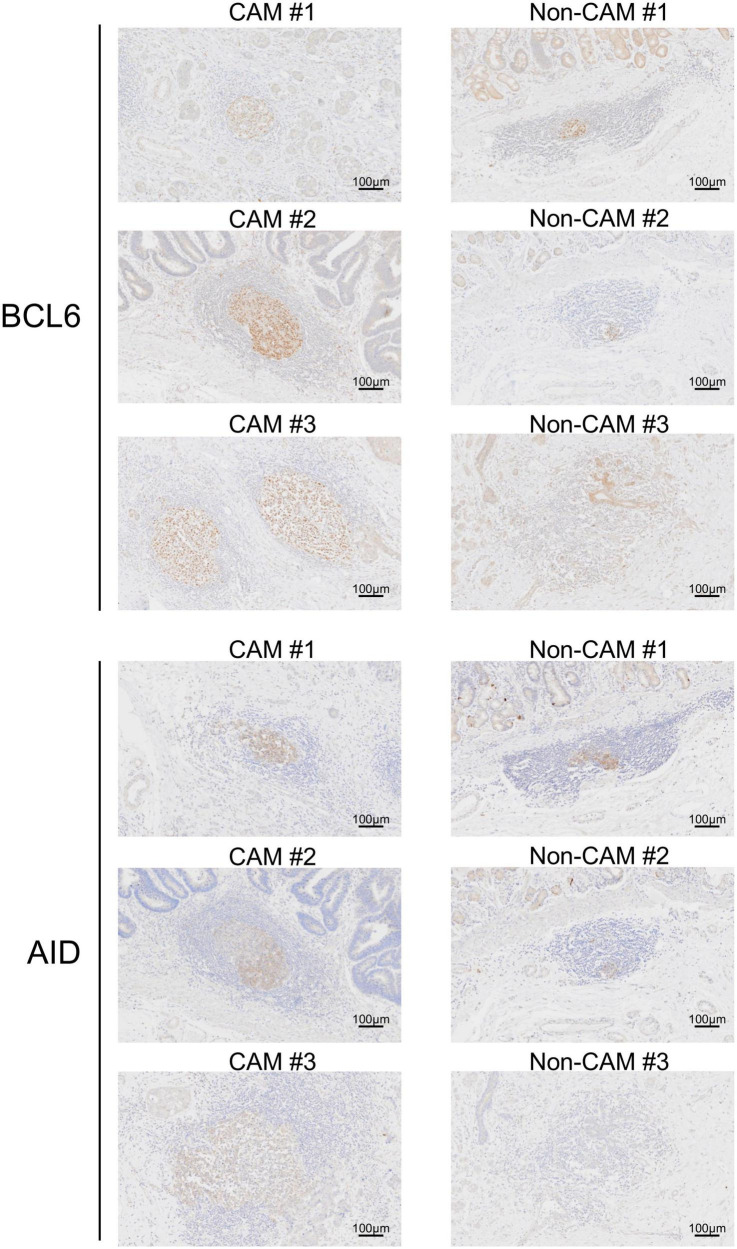
BCL6^+^ cells and AID^+^ cells in TLSs from CAM patients and non-CAM controls. Immunostaining for BCL6 or AID was conducted in representative TLSs from 3 CAM patients (CAM #1-#3) and 3 non-CAM controls (non-CAM #1-#3). Scale bar = 100 μm. CAM, cancer- associated myositis; TLSs, tertiary lymphoid structures; BCL6, B-cell lymphoma 6; AID, activation induced deaminase.

The cell densities of CD4^+^ T cells, CD8^+^ T cells, CD20^+^ B cells, PNAd^+^ HEV, CD23^+^ FDCs, CD138^+^ plasma cells, BCL6^+^ cells, and AID^+^ cells were evaluated in 20 TLSs from 3 CAM patients and 11 TLSs from non-CAM controls (Figure 5). We did not evaluate naïve and memory B cells separately since it has been reported that B cells in cancer tissues are mainly composed of memory B cells (29). Statistical analysis was not performed since the number of TLSs used for comparison varied among patients, resulting in the potential influence of data from particular patients on the overall results. There was no explicit difference in the density of CD4^+^, CD8^+^, and CD20^+^ cells; PNAd^+^ HEV; or CD138^+^ plasma cells between TLSs derived from CAM patients and those from non-CAM controls. On the other hand, the densities of CD23^+^ FDCs, BCL6^+^ cells, and AID^+^ cells in TLSs were numerically higher in TLSs derived from CAM patients than in those from non-CAM controls. Representative immunohistochemical staining of CD23, BCL6, and AID in TLSs is shown in Figures 6, 7.

**FIGURE 8:**
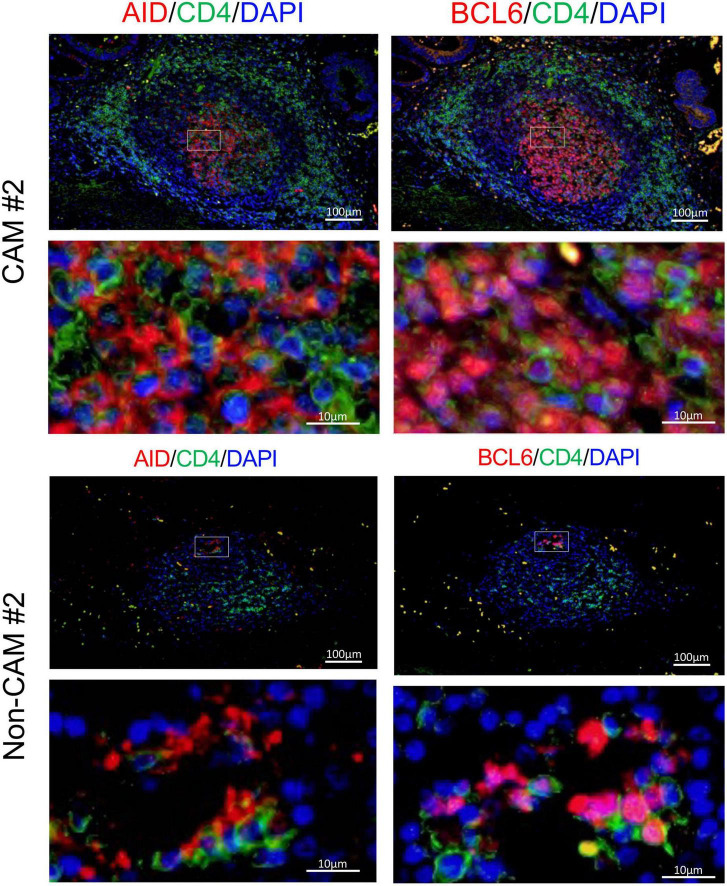
CD4^–^AID^+^ class-switched B cells and CD4^+^BCL6^+^ Tfh cells in TLS from a CAM patient and a non-CAM control. Immunofluorescent double-staining of CD4 and AID or BCL6 was conducted using sequential cancer sections. Representative images of the stomach cancer sections obtained from a CAM patient (CAM #2) and a non-CAM control (non-CAM #2) are shown. The staining of AID or BCL6 is shown in red, while CD4 staining is shown in green. Nuclei are counterstained with DAPI (blue). AID and BCL6 were stained in the cytoplasm and nucleus, respectively. The lower panel represents a high magnification image of an inset of the upper panel. Scale bar, 100 μm (upper) and 10 μm (lower). BCL6, B-cell lymphoma 6; TLSs, tertiary lymphoid structures; CAM, cancer-associated myositis; AID, activation induced deaminase; DAPI, 4’,6-diamidine-2’-phenylindole dihydrochloride; Tfh, T follicular helper.

**FIGURE 9:**
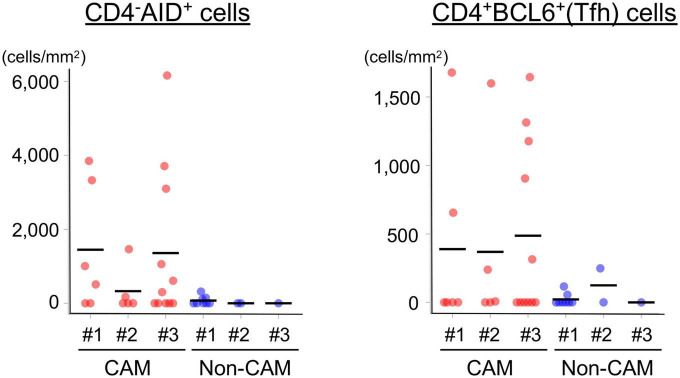
Density of CD4^–^AID^+^ class-switched B cells and CD4^+^BCL6^+^ Tfh cells in the TLSs from CAM patients and non-CAM controls. The density of CD4^–^AID^+^ class-switched B cells and CD4^+^BCL6^+^ Tfh cells in the TLSs from 3 CAM patients and 3 non-CAM controls was analyzed by computerized quantitative assessment using the bioimage analysis software QuPath. The horizontal lines indicate the mean. BCL6, B-cell lymphoma 6; TLSs, tertiary lymphoid structures; CAM, cancer-associated myositis; AID, activation induced deaminase; Tfh, T follicular helper.

Since AID^+^ cells and BCL6^+^ cells were mainly found in the lymphoid aggregates of the GC-like structure within TLSs, we further carried out immunofluorescent double-staining using sequential sections to assess their cell types and distribution (Figure 8). CD4^–^AID^+^ cells corresponding to class-switched B cells and CD4^+^BCL6^+^ Tfh cells were found in close proximity in the GC-like structure both in CAM patients and non-CAM controls. The density of CD4^–^AID^+^ class-switched B cells and CD4^+^BCL6^+^ Tfh cells was numerically greater in the CAM group than in the non-CAM control group (Figure 9).

## Discussion

In this case–control study, we demonstrated the intratumoral distribution of TLSs with a pronounced interaction of B cells and Tfh cells in the GC-like structure in CAM patients compared with non-CAM cancer controls whose demographic and cancer characteristics were matched with CAM cases. These findings suggest roles of acquired immune responses within the primary tumor site in developing CAM in patients with cancer.

Intratumoral TLSs are now recognized as a major player in the antitumor immune response since the presence and abundance of TLSs in the primary tumor lesion predicts favorable therapeutic responses and better progression-free survival (13, 14). Antitumor immunity is primed and enhanced by the presentation of neighboring tumor antigens by DCs, resulting in the maturation of T effector cells and memory B cells in the GC-like structure within TLSs (13). The association of TLSs with the improvement of overall survival in patients with cancer depends on their distribution within the tumor, maturation status, and cellular composition (30, 31). In fact, the induction of effective antitumor immunity and subsequent improved survival is associated with TLS distribution inside or at the border of cancer tissue, where the formation of tumor vessels facilitates the recruitment of immune cells into TLSs (10, 32–34). In this study, only one of 6 CAM patients enrolled in this study died during follow-up (case #5 with colorectal cancer), and it was difficult to identify histologic features of TLSs in the survivors. The predominant distribution of TLSs inside or at the border of cancer tissue in CAM patients suggests a potential relationship between an effective antitumor immune response and the emergence of CAM.

We failed to show a significant difference in the density and distribution of TILs between CAM patients and non-CAM controls, although there was a trend toward increased CD4^+^ T cells in the CT and IM areas in CAM patients. This finding can be explained by the predominant distribution of TLSs in the CT and IM areas in CAM patients. In addition, CD23^+^ FDCs, CD4^–^AID^+^ class-switched B cells, and Tfh cells were abundantly found in the GC-like structure of TLSs in CAM patients compared with non-CAM controls. In cancer patients, enriched B-cell signatures within TLSs are associated with a favorable treatment response to immune checkpoint inhibitors through activation of the antitumor immune response (35, 36). In this regard, CD23^+^ FDCs in TLSs possess Fcγ receptors and complement receptors and efficiently present antigens by binding of these receptors (13). Tfh cells in TLSs are specialized providers that help the organization of GC-like structures via a cognate interaction with B cells, resulting in the promotion of somatic hypermutation and isotypic switching of B cells and the subsequent generation of memory B cells and plasma cells producing high-affinity antibodies (37, 38). Finally, a recent study in patients with ovarian tumors found that TLSs facilitate coordinated antitumor immune responses involving not only high-affinity B cells but also cytolytic CD8^+^ T cells (35). Taken together, similar acquired immune responses might be activated in the primary tumor site in patients with CAM. Effective induction of adaptive immune responses against tumor cells plays a critical role in protection from cancer, while the upregulation of PD-1/PD-L1 plays an important role in evading tumor immune responses (39). Since PD-1/PD-L1 expression levels were positively correlated with expansion of TLSs/TILs in the cancer tissue (40), it is worth assessing a potential relationship between PD-1/PD-L1 expression and development of CAM in future studies.

Interestingly, mutations of genes, such as mismatch repair genes and the B-Raf proto-oncogene, in cancer cells are reported to promote the formation of TLSs (41). Somatic gene mutations and loss of heterozygosity of TIF1-γ, one of the major target autoantigens of CAM-associated MSAs (42), were found more frequently in cancer tissues of CAM patients with anti-TIF1-γ antibody than in those of non-CAM patients without anti-TIF1-γ antibody (43). In addition, it has been reported that the expression of autoantigens targeted by MSAs/MAAs, such as Jo-1 (histidyl-tRNA synthetase), Mi-2, U1-70kD, and Ku, is upregulated in cancer tissue (44). Based on these findings, one can hypothesize that immune responses elicited against self-antigens or modified self-antigens in cancer may be directed to the skin or skeletal muscles, leading to the development of CAM (45), although it was difficult to assess a potential relationship between histological features of TLSs and MSAs in this study due to the small number of patients analyzed.

Recently, much attention has been given to the roles of TLSs detected in the main target organs in the pathogenic process of many autoimmune diseases (46). These include TLSs in the synovium of patients with rheumatoid arthritis (RA), salivary glands of patients with Sjögren’s syndrome, and kidneys of patients with systemic lupus erythematosus. Interestingly, the formation of TLSs or TLS-like structures called inducible bronchus-associated lymphoid tissue in the lungs precedes the onset of RA in high-risk subjects who are positive for anti-cyclic citrullinated peptide antibodies (ACPAs) (47). Ectopic production of ACPAs within inducible bronchus-associated lymphoid tissue might be involved in the development of RA through cross-reaction to the citrullinated proteins expressed in the synovium (47, 48). Similarly, adaptive immune responses induced in cancer might cross-react with the autoantigens expressed on the skin and muscles (45). Lymphoid follicular structures similar to TLSs have been reported in the muscle tissue of patients with IIMs (49). It was interesting to compare TILs and lymphoid follicular structures between the tumor tissue and the affected tissues of CAM, including skeletal muscle and skin, but no case underwent muscle or skin biopsy in this study.

The strength of our study is the comparison between CAM patients and non-CAM patients using the case–control study design, in which background factors known to influence antitumor immune responses in cancer patients were matched between the groups. Another strength was the exclusion of patients who received immunomodulatory treatment before tumor resection surgery. These efforts certainly eliminated the potential influence of heterogeneous cancer factors and immunomodulatory treatment on immunologic features on histology, although major limitations were attributable to this study design. These included a small number of patients subjected to detailed analysis, leading to difficulty in performing appropriate statistical analysis. However, we believe that our findings warrant future studies recruiting a large number of cancer patients with and without CAM to explore a potential interaction between antitumor immunity and autoimmunity that elicits CAM.

## Conclusion

The adaptive immune response within TLSs in the primary tumor site might contribute to the development of CAM in selected patients with cancer. Our findings support the potential link between antitumor immunity and autoimmunity.

## Data availability statement

The raw data supporting the conclusions of this article will be made available by the authors, without undue reservation.

## Ethics statement

The studies involving human participants were reviewed and approved by Institutional Review Board of Nippon Medical School Hospital. The patients/participants provided their written informed consent to participate in this study.

## Author contributions

HK, TG, and MK: substantial contributions to the study conception and design, analysis and interpretation of data. HK, TG, SK, YO, MT, MS, and AS: substantial contributions to the acquisition of data. All authors drafting the article or revising it critically for important intellectual content and final approval of the version of the article to be published.

## Footnotes

1
https://www.aarda.org/diseaselist/

2
https://qupath.github.io
