# Supplementary material for: Tertiary lymphoid structures in the primary tumor site of patients with cancer-associated myositis: A case–control study
Source: Front Med (Lausanne). 2023 Jan 4;9:1066858. doi: 10.3389/fmed.2022.1066858 (PMC9845936; doi:10.3389/fmed.2022.1066858)
Supplement: Supplementary file 1 [file Data_Sheet_1.docx]

**Supplemental Figure S1. Patient flow for inclusion in this study**

CAM, cancer-associated myositis; IIM, idiopathic inflammatory myopathy; TLS, tertiary lymphoid structures

**Supplemental Table S1. Primary antibodies and antigen retrieval methods used for immunohistochemical analysis**

| Antigen | Antibody type | Species | Conjugate | Clone name | Company | Antibody titer | Antigen retrieval method |
| --- | --- | --- | --- | --- | --- | --- | --- |
| Immunohistochemistry | | | | | | | |
| CD3 | Polyclonal | Rabbit IgG | HRP | - | Agilent Technologies | 1:1 | High pH, MW 5min |
| CD4 | Monoclonal | Rabbit IgG1κ | HRP | 4B12 | Nichirei Bioscience Corporation | 1:1 | High pH, AC 20min |
| CD8 | Monoclonal | Mouse IgG1 | HRP | C8/144B | Nichirei Bioscience Corporation | 1:1 | Low pH, AC 20min |
| CD20 | Monoclonal | Mouse IgG2aκ | HRP | L26 | Agilent Technologies | 1:1 | High pH, MW 5min |
| DC-LAMP | Monoclonal | Rat IgG2a | HRP | 1010E1.01 | Novus Biologicals | 1:50 | Low pH, AC 15min |
| CD23 | Monoclonal | Mouse IgG1κ | HRP | 1B12 | Novocastra | 1:50 | Low pH, AC 15min |
| PNAd | Monoclonal | Rat IgMκ | HRP | MECA-79 | BD Pharmingen | 1:25 | Low pH, MW 20min |
| CD138 | Monoclonal | Mouse IgG1κ | HRP | M115 | Novocastra | 1:500 | Low pH, AC 20min |
| BCL6 | Monoclonal | Mouse IgG1 | HRP | GI191E/A8 | Cell Marque | 1:100 | High pH, AC 20min |
| AID | Monoclonal | Mouse IgG1κ | HRP | ZA001 | Thermo-Fisher | 1:100 | High pH, MW 15min |
| Immunofluorescence | | | | | | | |
| CD4 | Monoclonal | Rat IgG | - | SP35 | Abcam | 1:50 | High pH, MW 20min |
| BCL6 | Monoclonal | Mouse IgG1κ | - | PG-B6p | Agilent Technologies | 1:10 | High pH, MW 20min |
| AID | Monoclonal | Mouse IgG1κ | - | ZA001 | Thermo-Fisher | 1:100 | High pH, MW 20min |

HRP: horseradish peroxidase, DC-LAMP: dendritic cell lysosome-associated membrane glycoprotein, PNAd: peripheral node addressin, BCL6: B-cell lymphoma 6, AID: activation-induced cytidine deaminase, AC: autoclave, MW: microwave, NA: not available

**Supplemental Table S2. Clinical characteristics of 12 matched non-CAM controls**

|  | Non-CAM #1 | Non-CAM #2 | Non-CAM #3 | Non-CAM #4 | Non-CAM #5 | Non-CAM #6 | Non-CAM #7 | Non-CAM #8 | Non-CAM #9 | Non-CAM #10 | Non-CAM #11 | Non-CAM #12 |
| --- | --- | --- | --- | --- | --- | --- | --- | --- | --- | --- | --- | --- |
| Age at cancer resection surgery, years | 66 | 56 | 57 | 72 | 76 | 46 | 68 | 69 | 61 | 79 | 70 | 52 |
| Gender | Male | Female | Female | Male | Female | Female | Male | Female | Female | Male | Female | Female |
| Primary cancer site | Stomach | Stomach | Breast | Lung | Colorectum | Ovary | Stomach | Stomach | Breast | Lung | Colorectum | Ovary |
| Cancer histological type | Adeno carcinoma | Adeno carcinoma | Adeno carcinoma | Adeno carcinoma | Adeno carcinoma | Adeno carcinoma | Adeno carcinoma | Adeno carcinoma | Adeno carcinoma | Adeno carcinoma | Adeno carcinoma | Adeno carcinoma |
| TNM classification | pT1aN0M0 | pT1aN3M0 | pT2N2M0 | pT1N0M0 | pT3N0M0 | pT3cN1Mx | pT1bN0M0 | pT1bN0M0 | pT1N0M0 | pT2aN1M0 | pT3aN0M0 | pT3cNxM1 |
| Months from cancer diagnosis to last observation | 45 | 42 | 69 | 27 | 13 | 56 | 44 | 24 | 60 | 2 | 17 | 27 |
| Outcome at last observation | Dead | Alive | Alive | Alive | Alive | Alive | Alive | Alive | Alive | Alive | Alive | Alive |

CAM: cancer-associated myositis
